# Identification and Reproducibility of Plasma Metabolomic Biomarkers of Habitual Food Intake in a US Diet Validation Study

**DOI:** 10.3390/metabo10100382

**Published:** 2020-09-26

**Authors:** Ying Wang, Rebecca A. Hodge, Victoria L. Stevens, Terryl J. Hartman, Marjorie L. McCullough

**Affiliations:** 1 Department of Population Science, American Cancer Society, Atlanta, GA 30303, USA; becky.hodge@cancer.org (R.A.H.); vstevens311@gmail.com (V.L.S.); marji.mccullough@cancer.org (M.L.M.); 2 Department of Epidemiology, Rollins School of Public Health, Winship Cancer Institute, Emory University, Atlanta, GA 30322, USA; terryl.johnson.hartman@emory.edu

**Keywords:** untargeted metabolomics, food biomarker, FFQ, 24-h diet recalls, plasma

## Abstract

Previous metabolomic studies have identified putative blood biomarkers of dietary intake. These biomarkers need to be replicated in other populations and tested for reproducibility over time for the potential use in future epidemiological studies. We conducted a metabolomics analysis among 671 racially/ethnically diverse men and women included in a diet validation study to examine the correlation between >100 food groups/items (101 by a food frequency questionnaire (FFQ), 105 by 24-h diet recalls (24HRs)) with 1141 metabolites measured in fasting plasma sample replicates, six months apart. Diet–metabolite associations were examined by Pearson’s partial correlation analysis. Biomarker reproducibility was assessed using intraclass correlation coefficients (ICCs). A total of 677 diet–metabolite associations were identified after Bonferroni adjustment for multiple comparisons and restricting absolute correlation coefficients to greater than 0.2 (601 associations using the FFQ and 395 using 24HRs). The median ICCs of the 238 putative biomarkers was 0.56 (interquartile range 0.46–0.68). In this study, with repeated FFQs, 24HRs and plasma metabolic profiles, we identified several potentially novel food biomarkers and replicated others found in our previous study. Our findings contribute to the growing literature on food-based biomarkers and provide important information on biomarker reproducibility which could facilitate their utilization in future nutritional epidemiological studies.

## 1. Introduction

Self-reported diet assessment tools such as food frequency questionnaires (FFQs) have long been used to assess habitual diet in population studies. Such methods are subject to random and systematic measurement errors that could lead to underestimated diet–disease risk estimates and inconsistent findings in nutritional epidemiological studies [1]. Biomarkers are considered objective measures of diet and are not subject to the same measurement errors as self-reported diet, although other measurement errors may exist, and thus can complement or replace self-reported methods. Recovery dietary biomarkers can be used to estimate absolute intake (e.g., 24-h urinary nitrogen for protein intake) [2,3,4], and concentration biomarkers and predictive biomarkers can be used as stand-alone risk factors for disease outcomes, and to correct for measurement errors of a FFQ [5,6]. Although promising tools for diet assessment, the few established dietary biomarkers are primarily nutrient-based, and there is great potential and need for robust food-based biomarkers.

In recent years, metabolomics has been increasingly used to identify food-based biomarkers in human blood and urine samples [7]. It holds a great promise in nutritional epidemiology as an increasing number of food biomarkers have been identified and could be used to facilitate diet assessment in future research [1]. Several large metabolomics analyses conducted in cohort studies with biospecimens have identified biomarkers of habitual food intakes [8,9,10,11,12,13,14] or dietary patterns [15,16]. In our previous metabolomics analysis of 91 food groups and 1186 serum metabolites among 1369 nonsmoking postmenopausal women in the Cancer Prevention Study II (CPS-II) Nutrition Cohort, we identified 379 diet–metabolite associations with 199 metabolites as putative food biomarkers of 42 food groups/items (one metabolite could be biomarker of multiple food groups/items) [8]. Many of the biomarkers were previously identified in population and/or intervention studies, and thus were validated in our study (e.g., stachydrine for citrus fruit intake). Novel biomarkers with high sensitivity and specificity for the correlated food intake included alliin for garlic intake and dopamine 3-O-sulfate for banana intake. These newer biomarkers need to be replicated across diverse populations.

One concern of using these biomarkers in population studies is that one-time measurement may poorly reflect long-term status [17]. Large day-to-day variation in certain metabolite levels due to measurement and random errors could lead to underestimation of diet–disease associations if only measured once. Therefore, it is important to assess biomarker reproducibility over time to determine if one-time measurement is sufficient to capture usual exposure.

In the Diet Assessment Sub-study (DAS) from the Cancer Prevention Study-3 (CPS-3) cohort, where diet and fasting blood samples were measured twice six months apart, we aimed to (1) replicate and identify metabolites associated with individual food groups/items using untargeted metabolomic profiling, and (2) to assess the reproducibility of identified metabolites over six months.

## 2. Results

### 2.1. Participant Characteristics

Characteristics of the study population are shown in Table 1. Among the 671 participants in the DAS, 60.1% were white, 24.7% were black, 15.2% were Hispanic. The majority (65.1%) were female. The mean age was 52.3 ± 9.5 years.

### 2.2. Fasting Plasma Metabolites Correlated with Habitual Food Intake Assessed by FFQ and 24-h Diet Recalls (24HRs)

We identified a total of 677 food–metabolite associations (Table S1). A total of 601 associations were found using the post-FFQ (*p* < 4.33 × 10^–7^ and |r| > 0.2, Table S2), and 395 associations were found using the average 24HRs (*p* < 4.17 × 10^–7^ and |r| > 0.2, Table S3); 238 plasma metabolites were associated with 74 food groups or items assessed using either the FFQ or 24HRs. The majority of the diet-related metabolites (n = 238) were xenobiotics (n = 67; 28%), unknowns (n = 63; 26%) and lipids (n = 62; 26%); the rest were amino acids (n = 28; 12%), cofactors and vitamins (n = 10; 4%), peptides (n = 2; 1%), carbohydrates (n = 2; 1%), nucleotides (n = 1; 0.4%) and partially characterized molecules (n = 3; 1%).

The AUCs were calculated to inform the predictive accuracy of the diet-related metabolites. The top three most predictive metabolites (according to FFQ, if less than three then according to 24HRs) for each of the 74 food groups or items are shown in Table 2. For most food groups, the most predictive metabolite also had the highest |r|.

#### 2.2.1. Fruits

We identified 51 food–metabolite associations for 13 fruit groups or items estimated either from FFQ or 24HRs, including10 for avocado, 1 for apples or pears, 2 for apples (24HRs only), 12 for total citrus fruits and juices, 7 for oranges, 5 for orange juice, 2 for grapefruit, 1 for watermelon, 1 for cantaloupe, 3 for berries, 4 for blue berries, 1 for raspberry, 2 for peaches and plums; 42 associations were observed for 10 groups/items from the FFQ and 33 associations for 8 groups/items from the 24HRs. The strongest associations were found for 3-hydroxystachydrine (r = 0.50, AUC = 94%) and stachydrine (r = 0.50, AUC = 93%) with total citrus fruit and juice intake assessed by the FFQ. Notably, 4-allylphenol sulfate is correlated with intakes of apples or pears (r = 0.2, AUC = 79%) and blueberries (r = 0.22, AUC = 82%) assessed by the FFQ.

#### 2.2.2. Vegetables

We identified 75 associations for 16 vegetable groups or individual vegetables, with 53 associations for 14 groups/items from the FFQ, and 38 associations for 8 groups/items from the 24HRs. Specifically, we identified 1 metabolite for tomatoes, 3 for asparagus, 3 for beans, 19 for all soy products, 7 for fermented soy products, 5 for soy milk, 1 for soy protein powder, 8 for cruciferous vegetables, 4 for leafy greens, 1 for iceberg or head lettuce, 1 for peppers, 5 for mushrooms (24HRs only), 3 for allium vegetables, 3 for onions, 10 for garlic and 1 for garlic powder. Of these, the strongest association was seen for an unknown metabolite X-16649 with soy products assessed by the 24HRs (r = 0.37, AUC = 75%).

#### 2.2.3. Grains

We identified 18 food–metabolite associations for 5 grain groups/items (4 for total whole grains, 1 for whole grain bread, 5 for whole grain cereals, 5 for corn products and 3 for refined grains), with 15 associations using FFQ, and 8 using 24HRs. An unknown metabolite X-21752 was the most predictive metabolite for total whole grains (r = 0.31, AUC = 89%) and whole grain cereals (r = 0.42, AUC = 87%) assessed using the FFQ.

#### 2.2.4. Proteins

We identified 181 diet–metabolite associations for 11 protein foods (2 for egg, 31 for red meat, 30 for processed meat, 46 for poultry, 17 for total fish, 16 for dark fish, 6 for shellfish, 7 for total nuts, 12 for peanuts, 7 for other nuts and 7 for seeds); 164 associations for 11 groups/items were identified using the FFQ and 99 associations for 10 groups/items using the 24HRs. The strongest association was between X-13835 and FFQ-assessed poultry intake (r = 0.54, AUC = 85%).

#### 2.2.5. Dairy/Dairy Alternatives

There were 41 diet–metabolite associations for 4 dairy/dairy alternative groups (6 for milk, 3 for almond milk or rice milk, 14 for total cheese, 18 for cream); 39 associations were found using the FFQ, and 13 using the 24HRs. The strongest association was between X-11381 and milk (r = 0.33, AUC = 84%). Almond milk or rice milk was a new line item on the CPS-3 FFQ. The only metabolite that had a positive association with almond milk (X-24475) was also associated with intake of other nuts. All the cheese-related metabolites were fatty acids and sphingomyelins. All of the 18 metabolites associated with cream intake, a majority being xenobiotics, also were associated with coffee intake, indicating that the two were commonly consumed together and these biomarkers should not be considered as specific biomarkers for cream intake.

#### 2.2.6. Fats and Oils

We identified 16 associations for creamy salad dressing (n = 12), oil and vinegar salad dressing (n = 2) and olive oil (n = 2), and 15 were found using the FFQ and 9 found by the 24HRs.

#### 2.2.7. Alcohol

Using either instrument, we identified 172 associations for alcohol, including 58 for total alcohol, 19 for beer, 44 for wine, 39 for red wine, 8 for white wine and 4 for liquor. Using the FFQ, 160 associations were found, and using the 24HRs, 102 associations were found. Ethyl alpha-glucopyranoside was the most predictive metabolite for total alcohol (r = 0.52, AUC = 95%) and individual types of alcohol (AUC ranging from 74% for white wine to 91% for total wine) assessed using the FFQ. Ethyl glucuronide was the second most predictive metabolite for total alcohol.

#### 2.2.8. Beverages

There were 80 associations for beverages, including 33 for total coffee, 34 for caffeinated coffee, 2 for decaffeinated coffee, 4 for total tea, 1 for green tea, 3 for black tea, 2 for herbal tea and 1 for diet beverages, with 77 found from the FFQ and 71 from 24HRs. Quinate and the unknown X-21442 were the most predictive metabolites for total coffee consumption (r = 0.77, AUC = 99% and r = 0.81, AUC = 99%, respectively). The majority of metabolites correlated with total coffee and caffeinated coffee were involved in xanthine and benzoate metabolism. For tea consumption, theanine was the most predictive biomarker, slightly stronger for black tea than for green tea, and strongest for total tea (r = 0.40, AUC = 86%). Acesulfame was associated with diet beverage consumption (r = 0.42, AUC = 82%).

#### 2.2.9. Miscellaneous

The remaining 43 associations were found for miscellaneous foods, 10 for French fries, 3 for ice cream, 10 for chips, 6 for chocolate candies, 7 for dark chocolate, 1 for energy/protein bars, 3 for soy sauce and 3 for artificial sweeteners. Several xanthine metabolites that were correlated with coffee intake were also correlated with chocolate intake, including theobromine, 3-methylxanthine and 7-methylxanthine. In addition to acesulfame that was correlated with diet beverages, two more metabolites—saccharin and erythritol—were associated with overall artificial sweetener intake.

### 2.3. Reproducibility of the Identified Food Metabolites

Of the 238 metabolites that were significantly associated with food groups/items identified via FFQ or 24HRs, the median ICC calculated using duplicate samples over six months was 0.56 (interquartile range: 0.46–0.68). By super pathway, the median ICC ranged from 0.39 for carbohydrates to 0.69 for cofactors and vitamins.

**Figure 1:**
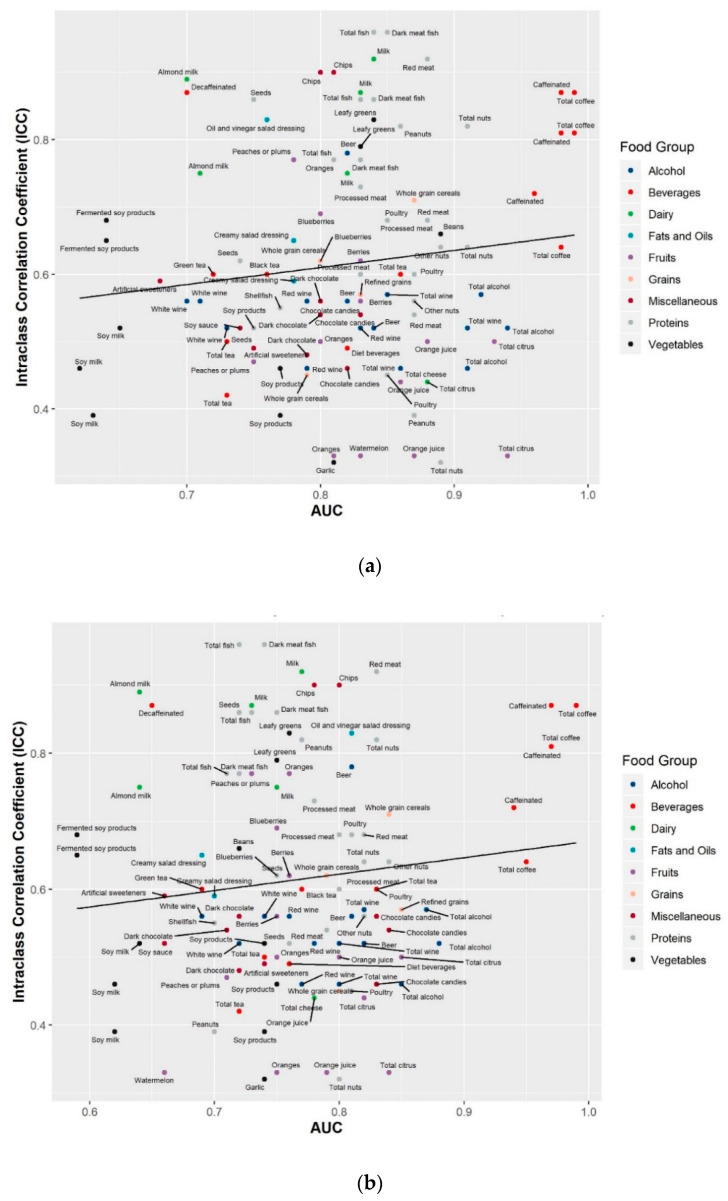
Metabolite prediction accuracy for food intake by metabolite reproducibility for the top three predictive metabolites of 74 food groups/items in the Cancer Prevention Study-3 Diet Assessment Sub-study. (**a**) Top three predictive metabolites for food intake assessed using the food frequency questionnaire; (**b**) top three predictive metabolites for food intake assessed using the average of 24-h diet recalls. Prediction accuracy was assessed by area under the curve (AUC) from the receiver operating characteristic curve, which indicates how well a metabolite could discriminate top quartile from bottom quartile intake of a food group/item. Reproducibility was assessed by intraclass correlation coefficients (ICCs), calculated as the ratio of between-person variance to the total variance among participants with repeated blood metabolic profiles measured six months apart.

Combining information on both accuracy (AUC) and reproducibility (ICC) over time can indicate if a biomarker is reliable to be used in future epidemiological studies. The combined information on AUC and ICC for the top three metabolites of the 74 food groups/items are shown in Figure 1. Biomarkers in the upper right corner with both high AUC and ICC are considered reliable, while those in the lower left corner with the low AUC and ICC are less reliable. AUCs obtained from 24HRs were generally lower than those from the FFQ. In the present study, such reliable biomarkers were seen for several food groups/items including fish, milk, meat, nuts, coffee, leafy greens, oranges and whole grain cereals. Biomarkers with high AUCs but low ICCs might be useful in short-term studies to monitor dietary intake compliance but may require more than one measurement to capture long-term levels.

## 3. Discussion

In this yearlong diet validation study with repeated measures of diet using both FFQ and 24HRs and two measures of fasting plasma metabolic profiles approximately 6 months apart, we replicated many food–metabolite associations that were found in other studies, and identified several potentially novel food biomarkers. More associations were found via FFQ than via 24HRs. Reproducibility of the 238 identified metabolites was acceptable for a large proportion, with 38% of metabolites with an ICC > 0.6. Our findings contribute to the literature on food-based biomarkers and provide important information on the reproducibility of the biomarkers which could facilitate their utilization in future nutritional epidemiological studies.

Generally, we identified more food–metabolite associations using the FFQ than using the 24HRs. Additionally, the biomarker AUCs were higher in general using the FFQ than using the 24HRs. In other words, the identified biomarkers predict dietary intake assessed via the FFQ better than that via the 24HRs. Even though the repeated measurements using 24HRs are considered a superior method of assessing the true intake in the validation study, the FFQ is designed to capture usual food intake in the past 12 months. That metabolites correlated better with the FFQ than the average 24HRs may indicate that the biomarkers reflect a long-term status of dietary intake. We observed a greater number of associations in the current study than in our previous study in the CPS-II Nutrition Cohort [8], probably because in the CPS-3, the FFQ was collected in closer proximity to blood draw (as part of the validation study), and using an average of two blood samples likely better captured usual metabolite levels during the year.

We replicated five metabolites that had been correlated with total citrus fruits and juices or orange juice in the CPS-II Nutrition Cohort [8]. Stachydrine—the strongest biomarker of total citrus fruits and juices—was first identified in an acute feeding study [18] and then validated as a biomarker of habitual citrus fruit intake in several cross-sectional datasets [9,10,11,12,13,19,20,21] including our previous metabolomics study in the CPS-II Nutrition Cohort [8]. Among the food biomarkers we identified in the CPS-3 DAS but not in CPS-II Nutrition Cohort, 4-allylphenol sulfate that is associated with apple/pear and blueberry intake is a nonspecific microbial metabolite of polyphenols [22], and has been reported as a biomarker of pears in a randomized trial [23]. Among the 75 vegetable–metabolite associations, 14 were found in the CPS-II Nutrition Cohort [8]. Notably, we replicated ergothioneine as a putative biomarker of mushroom intake, and several metabolites such as alliin, N-acetylalliin and S-allylcysteine as biomarkers of garlic intake. We previously found S-methylcysteine sulfoxide as a biomarker of cruciferous vegetable intake [8] which was also reported in the Prostate, Lung, Colorectal and Ovarian (PLCO) cohort [24]. In the present study, we found S-methylcysteine, the biological precursor of S-methylcysteine sulfoxide to be associated with cruciferous vegetable intake. Among the food–metabolite associations not found in the CPS-II Nutrition Cohort, S-methylcysteine and pipecolate were reported as useful dry bean biomarkers in both human and mouse studies [25]; genistein sulfate and 4-ethylphenyl sulfate are biomarkers for soy product intake. 4-ethylphenyl sulfate is a uremic toxin produced by gut bacteria, and its association with soymilk has been reported in a cohort of female twins [9].

We identified several new biomarkers for whole grain products such as 2,6-dihydroxybenzoic acid, 2-aminophenol sulfate and 2-acetamidophenol sulfate compared with our previous study in the CPS-II Nutrition Cohort [8]. 2,6-dihydroxybenzoic acid is a phenolic acid, also known as γ-resorcylic acid, which was identified as a marker for a high dietary fiber intake in an intervention study [26]. It is possible that 2,6-dihydroxybenzoic acid was derived from alkylresorcinols or lignans through a speculated microbial enzyme not yet identified in humans [26]. 2-acetamidophenol sulfate (HPAA sulfate) and 2-aminophenol sulfate are benzoxazinoid metabolites that were previously found as biomarkers of whole grain intake in urine [27]. 2-aminophenol sulfate was also found to be elevated in plasma after high dietary fiber intake [26].

Most of the metabolites associated with egg, meat and poultry intake are amino acids and lipids, especially plasmalogens. Two novel biomarkers of red meat are xenobiotics 3-bromo-5-chloro-2,6-dihydroxybenzoic acid and 3,5-dichloro-2,6-dihydroxybenzoic acid, which were also correlated with milk intake in the present study. We replicated three metabolites that have been associated with habitual consumption of fish and shellfish in our previous study [8], including 3-carboxy-4-methyl-5-propyl-2-furanpropanoate (CMPF), hydroxy-CMPF (previously known as X-02269) and docosahexaenoate (DHA; 22:6 n3). The most predictive metabolite hydroxy-CMPF (X-02269) for total fish was also reported in the TwinsUK cohort [9] and a US cohort [13]. Among the five metabolites that correlated with nut intake, tryptophan betaine and 4-vinylphenol sulfate were also reported in similar cross-sectional studies [8,10,12,13].

In our previous study [8], ethyl glucuronide was the most predictive metabolite of all types of alcohol and is metabolized directly from ethanol in the liver by UDP-glucuronosyltransferases [28]. In the present study, the most predictive metabolite of alcohol was ethyl alpha-glucopyranoside (previously known as X-24293), which is a glycoside found in Japanese rice wine and might be used as a functional food or cosmetic material [29]. For wine consumption (total and red but not white wine), we replicated the potential biomarker 2,3-dihydroxyisovalerate, an intermediate metabolite produced by yeast during wine fermentation [30]. We replicated 26 metabolites as biomarkers of total coffee intake [8], including quinate, the highly predictive unknown metabolite X-21442, several caffeine metabolites (e.g., 1-methylxanthine, 1,3-dimethylurate, 1,7-dimethylurate, 1,3,7-trimethylurate) and other metabolites. Chlorogenic acid, an abundant natural polyphenol, is found in high concentration in coffee. During the roasting process, chlorogenic acid is broken down to quinate and caffeic acid. In both the CPS-II Nutrition Cohort and CPS-3 DAS, quinate was among the top predictive biomarkers of caffeinated and decaffeinated coffee. Previous animal studies showed chlorogenic acid and related compounds exert antiviral [31] and anticarcinogenic effects [32,33]. Future human studies need to investigate these biomarkers with disease outcomes directly or through mediation analyses. For tea consumption, we replicated that theanine was the most predictive biomarker for total tea, green tea and black tea consumption.

As discussed above, our studies (both in CPS-3 DAS and our prior research in the CPS-II Nutrition Cohort [8]) and others have identified many biologically plausible, putative food biomarkers using metabolomics, which highlights the importance of this technology in identifying dietary biomarkers. Moving forward, more research is needed to determine the use of these putative biomarkers in diet assessment. One important step is to develop calibration equations in controlled feeding studies, so that the biomarkers may be used to correct self-reported dietary intake [1]. Urinary recovery biomarkers have been used to calibrate energy and protein intakes and showed improved diet–disease associations compared with uncalibrated data [34]. Lampe et al. also evaluated blood concentration biomarkers in a feeding study of postmenopausal women and suggested that they perform as well as recovery biomarkers and, therefore, can be used to correct self-reported dietary intake data in future studies [35]. Cross-sectional studies such as the present study provide important information as one could examine multiple foods simultaneously and determine if a metabolite is correlated with multiple foods. Among the identified metabolites, many may not be optimal food biomarkers if they are not specific to certain foods or if they are synthesized endogenously, because their levels will be influenced by other characteristics.

One concern of using the metabolomic biomarkers in epidemiological studies is that one-time measurement is subject to short-term variation and may not represent long-term status. Large within-person variation compared to between person variation in metabolite levels can contribute to measurement errors that would result in underestimated disease risk estimates. An ICC, the ratio of between-person variance to total variance, is a good indicator of metabolite reproducibility. High ICCs indicate large between-person variation relative to the total variation, such as biomarkers for fish, milk, meat and coffee. Low ICCs indicate large within-person variation relative to the total variation. However, a low ICC does not necessarily exclude the metabolite from being used as a dietary biomarker in all circumstances. The low ICCs observed in the present study could be due to the infrequency of consumption of certain foods e.g., soy products, and could also be due to the seasonal variation in consumptions of certain fruits and vegetables, as one of the purposes of the CPS-3 DAS was to capture seasonal variation in blood biomarkers by collecting the samples six months apart. If collected a year apart, we would expect to see higher ICCs for many biomarkers of the foods that are consumed seasonally. A few previous studies examined the reproducibility of metabolites over a period, although did not focus on diet related biomarkers [36,37]. Floegel et al. [36] investigated the ICCs of 163 fasting serum metabolites over a 4-month period and found that the median ICC was 0.57 (vs. median ICC of 0.56 over six months in the present study). Carayol et al. [37] found a median ICC of 0.70 among 158 metabolites measured in fasting plasma samples over a 2-year period. They also found that the ICCs were higher for metabolites measured in fasting samples than in nonfasting samples, although Sampson et al. [17] found that fasting is not a major source of variation in metabolite levels in population studies. Therefore, one-time measurement is likely sufficient for many of the metabolites with high reproducibility.

The present study has several strengths. Its large sample size and comprehensive dietary and metabolomic data allowed us to explore a large number of diet–metabolite associations simultaneously which is more efficient than feeding studies and can provide information on the specificity of the biomarkers. Furthermore, the repeated measurements of blood samples enabled us to test biomarker reproducibility over 6 months. Our findings confirmed many previously identified food biomarkers and identified new metabolites for further testing. Reproducibility of food-based biomarkers is largely unknown in the field but very important to inform the application of such biomarkers in etiologic analyses. Large within-person variation in the biomarker over time is a major source of measurement error that could lead to underestimated diet–disease associations and inconsistent findings. Additional feeding studies are needed to test the dose–response relationships between food intake and the identified biomarkers to further confirm their validity for future use.

## 4. Materials and Methods

### 4.1. Study Population

The Diet Assessment Sub-study (DAS) was a one-year study among 745 men and women enrolled in the CPS-3 cohort, designed to evaluate the validity of the CPS-3 FFQ. Briefly, CPS-3 is a large prospective cohort study of 303,682 adults aged 30–65 residing in 35 states in the United States, plus the District of Columbia and Puerto Rico, who were enrolled between 2006 and 2013 [38]. At enrollment, participants provided a blood sample, had their waist circumference measured and completed an enrollment survey. They were also asked to complete a comprehensive baseline survey that assessed demographic, lifestyle and medical information. Follow-up questionnaires were sent in 2015 to those who completed the baseline survey after enrollment (N = 254,650) to update lifestyle and medical information and to assess diet using the CPS-3 FFQ for the first time.

The CPS-3 DAS was designed to evaluate the validity and reproducibility of the newly modified CPS-3 FFQ over a year. CPS-3 participants living in 5 regions defined by Quest Diagnostics business units (Atlanta, GA, USA; Dallas, TX, USA; Auburn Hills, MI, USA; West Hills, CA, USA; San Jose, CA, USA) were invited to participate in DAS. Participants were asked to complete the 2015 follow-up survey (to serve as the first FFQ), six telephone-administered 24HRs throughout the year, provide two fasting blood and two 24-h urine samples and complete the post-FFQ at the end of the study. The six 24HRs aimed to include four weekdays and two weekend days, with a goal of obtaining two 24HRs per “trimester”; we aimed to collect one 24HR within a week prior to the fasting blood draw. Blood and urine samples were collected six months apart to capture seasonal variation.

A total of 745 men and women completed both FFQs and the first 24HR, meeting the minimum criteria to remain in the DAS. For the metabolomics analysis, we excluded participants who completed less than three 24HRs (n = 2), had poor post-FFQs (n = 20; defined as missing 2 or more sections, an entire page, >100 line items or with daily energy intake <800 or >4500 kcal for men, and <600 or >3800 kcal for women) or had no blood sample (n = 1). We further excluded current smokers (n = 21), those whose body weight was missing at both blood draw appointments (n = 3) or weight change was >20 lbs between blood draws (n = 14) and pregnant women (n = 13). A total of 671 men and women were included in this plasma metabolomics analysis. Those with two blood draws (n = 644) were included in the metabolomic reproducibility analysis (Figure S1). The CPS-3 DAS protocol was approved by the Emory University (Atlanta, GA, USA) Institutional Review Board.

### 4.2. Diet Assessment

Diet was assessed using the newly modified CPS-3 FFQ as described elsewhere [39]. Briefly, the Willett FFQ [40,41] was modified for the CPS-3 study population, to capture racial/ethnic and geographic diversity of the cohort. Modifications to the FFQ were informed through telephone-administered 24HRs, analyses of NHANES 2009–2010 and focus groups [39]. The final modified FFQ included 191-line items. Only the post-FFQ was used to assess dietary intake in the present study. We defined 101 food groups/items from the FFQ as shown in Supplemental Table S1, similar to what we defined in the CPS-II Nutrition Cohort [8]. Comparable food groups were derived from the 24HRs to match those from the FFQ. We also created a few food groups using the 24HRs that are not asked (e.g., mushroom) or asked in combination with other foods (e.g., apples) on the FFQ. A total of 105 food groups/items were derived from the 24HRs.

### 4.3. Blood Collection and Processing

Participants were instructed to make an appointment with a Quest Patient Service Center to have fasting blood drawn on the morning of the visit. Participants were asked to follow their usual diet except during the 8-h fasting period before the appointment. A total of 40 mL of fasting blood was collected using 5 EDTA tubes for plasma collection, and 4 serum separator tubes for serum collection. Blood samples were refrigerated and transferred to a Quest Diagnostics regional processing laboratory where they were fractionated by centrifugation and aliquoted into 9 vials. All aliquots of blood were frozen and shipped on dry ice to an off-site biorepository (Fisher Biorepositories, Inc., Frederick, MD, USA) for long term storage in the vapor phase of liquid nitrogen.

### 4.4. Metabolomics Analysis

Metabolomic profiling was conducted by Metabolon, Inc. (Durham, NC, USA) using ultrahigh performance liquid chromatography-tandem mass spectrometry (UPLC-MS/MS) described elsewhere [42]. Briefly, plasma samples were treated with methanol to precipitate proteins. Four sample fractions were dried and reconstituted in different solvents for measurement under four different platforms. These platforms consisted of two separate reverse phase UPLC-MS/MS methods with positive ion mode electrospray ionization (ESI), one reverse phase UPLC-MS/MS method with negative ion mode ESI and one hydrophobic interaction chromatography UPLC-MS/MS with negative ion mode ESI. Individual metabolites were identified by comparison with a chemical library maintained by Metabolon that comprises more than 3300 commercially available purified standard compounds and recurrent unknown entities, based on retention index, mass to charge ratio and chromatographic data.

A total of 1368 metabolites were detected in the fasting plasma samples. Metabolites that were below the detection limit in >90% of the samples were excluded (n = 131). For the remaining metabolites, missing values were assigned the minimum detection value. To correct the day-to-day variation from the platform, each metabolite was divided by its daily median. Duplicates of 60 participant samples were used as quality controls to assess inter- and intrabatch variation. Interclass correlation coefficients (ICCs) were calculated among the quality control samples to test the reproducibility of the platform. Metabolites with ICC < 0.5 were further excluded from the analysis, leaving 1141 for food–metabolite analysis. Of the 1141 included metabolites, the median technical ICC was 0.87, with an interquartile range of 0.77 to 0.93, suggesting a very high reproducibility of the platforms.

### 4.5. Statistical Analysis

Metabolite and food variables were generalized log transformed [43] and autoscaled before all analyses. Pearson’s partial correlation was used to determine the food–metabolite associations, controlling for age (continuous), gender, race/ethnicity (white, black, Hispanic), education (no college, college graduate, graduate school, unknown), smoking status (never, former), physical activity (metabolic equivalent hours per week (MET-h/wk): <5, 5–<10 or missing, 10–<15, ≥15), body mass index (kg/m^2^, continuous), ethanol intake (g/d, continuous; except for alcohol containing items) and energy intake (kcal/d, continuous). Associations were considered statistically significant if *p* values were less than the Bonferroni-corrected threshold (0.05/1141/101 = 4.33 × 10^–7^ for FFQ, 0.05/1141/105 = 4.17 × 10^– 7^ for 24HRs). To select more meaningful associations, we further required that the absolute values of the correlation coefficient (|r|) were greater than 0.2.

Putative dietary biomarkers were further evaluated for predictive accuracy of discriminating high consumers (top quartile) from low consumers (bottom quartile), assessed using the area under the curve (AUC) calculated from the receiver operating characteristic (ROC) curve using R package pROC [44]. We considered AUC < 0.7 to be low, 0.7–<0.8 to be moderate and ≥0.8 to be high.

The reproducibility of the identified food-related metabolites over six months was assessed using ICCs. ICCs were calculated as the ratio of between-person variance to the total variance among participants with repeated measures of blood metabolic profiles. Between-person variance was estimated from a random effects model where participants were modeled as a random variable. ICCs >0.6 were considered good and >0.75 considered excellent.

## 5. Conclusions

In conclusion, in this large and comprehensive analysis of habitual diet and fasting plasma metabolic profiles in a free-living population of racially/ethnically diverse men and women, we identified several potentially novel food biomarkers and replicated others found in previous studies. Our findings contribute to the growing literature on food-based biomarkers and provide important information on the reproducibility of the biomarkers which could facilitate their utilization in future nutritional epidemiological studies.

## Figures and Tables

**Table 1:** Characteristics of participants in the Cancer Prevention Study-3 Diet Assessment Sub-study ^1^.

Characteristics	Men (n = 234)	Women (n = 437)
Age (year)	52.4 ± 10.0	52.2 ± 9.2
Race/ethnicity		
White	147 (62.8)	256 (58.6)
Black	42 (17.9)	124 (28.4)
Hispanic	45 (19.2)	57 (13.0)
BMI at pre-FFQ (kg/m^2^)	27.5 ± 5.4	27.7 ± 6.6
Education		
<College	40 (17.1)	108 (24.7)
College	82 (35.0)	144 (33.0)
≥Graduate school	103 (44.0)	170 (38.9)
Unknown	9 (3.8)	15 (3.4)
Smoking status		
Never	181 (77.4)	347 (79.4)
Former	53 (22.6)	90 (20.6)
Recreational physical activity (MET-h/wk)		
0–<5	44 (18.8)	124 (28.4)
5–<10 ^2^	74 (31.6)	147 (33.6)
10–<15	50 (21.4)	78 (17.8)
≥15	66 (28.2)	88 (20.1)
Ethanol intake (g/d)	10.3 ± 13.9	7.0 ± 11.5
Energy from post-FFQ (kcal/d)	2136 ± 690	2007 ± 609
Average energy intake from 24HRs (kcal/d)	2214 ± 583	1730 ± 414

Abbreviations: BMI, body mass index; 24HR, 24-h diet recall; FFQ, food frequency questionnaire; MET-h, metabolic equivalent hour. ^1^ Values are mean ± standard deviation for continuous variables, and frequency (%) for categorical variables. ^2^ Includes missing.

**Table 2:** Top three predictive metabolites for 74 food group/item assessed using the CPS-3 FFQ and average of 24-h diet recalls in the Cancer Prevention Study-3 Diet Assessment Sub-study ^1^.

Food Group/Items	Biochemical Name ^2^	Super Pathway	Post-FFQ			Average Dietary Recalls			ICC ^3^
			R	*p* Value	AUC	R	*p* Value	AUC	
**FRUITS**									
Avocado	X-11315		**0.22**	**2.43 × 10^−8^**	0.82	0.18	6.24 × 10^−6^	0.74	0.64 (0.59, 0.68)
	X-24475		**0.21**	**9.21 × 10^−8^**	0.82	0.13	1.07 × 10^−3^	0.73	0.56 (0.51, 0.61)
	X-11858		**0.24**	**2.51 × 10^−10^**	0.82	0.15	1.64 × 10^−4^	0.73	0.52 (0.47, 0.58)
Apples or pears	4-allylphenol sulfate	Xenobiotics	**0.20**	**1.63 × 10^−7^**	0.79				0.44 (0.38, 0.50)
Apples ^4^	4-allylphenol sulfate	Xenobiotics				**0.21**	**3.04 × 10^−8^**	0.70	0.44 (0.38, 0.50)
	β -cryptoxanthin	Cofactors and Vitamins				**0.20**	**1.58 × 10^−7^**	0.70	0.77 (0.74, 0.80)
Total citrus fruits and juices	3-hydroxystachydrine *	Xenobiotics	**0.50**	**1.37 × 10^−43^**	0.94	**0.47**	**4.27 × 10^−38^**	0.84	0.33 (0.27, 0.40)
	stachydrine	Xenobiotics	**0.50**	**1.31 × 10^−43^**	0.93	**0.46**	**3.27 × 10^−36^**	0.85	0.50 (0.44, 0.55)
	N-methylproline	Amino Acid	**0.38**	**7.98 × 10^−24^**	0.88	**0.39**	**3.38 × 10^−25^**	0.82	0.44 (0.38, 0.51)
Oranges	β -cryptoxanthin	Cofactors and Vitamins	**0.30**	**3.05 × 10^−15^**	0.81	**0.33**	**1.07 × 10^−17^**	0.76	0.77 (0.74, 0.80)
	3-hydroxystachydrine *	Xenobiotics	**0.31**	**1.13 × 10^−15^**	0.81	**0.27**	**1.38 × 10^−12^**	0.75	0.33 (0.27, 0.40)
	stachydrine	Xenobiotics	**0.30**	**7.26 × 10^−15^**	0.80	**0.25**	**3.91 × 10^−11^**	0.75	0.50 (0.44, 0.55)
Orange juice	stachydrine	Xenobiotics	**0.35**	**6.46 × 10^−21^**	0.88	**0.34**	**1.28 × 10^−19^**	0.80	0.50 (0.44, 0.55)
	3-hydroxystachydrine *	Xenobiotics	**0.35**	**7.40 × 10^−21^**	0.87	**0.33**	**1.19 × 10^−18^**	0.79	0.33 (0.27, 0.40)
	N-methylproline	Amino Acid	**0.30**	**3.41 × 10^−15^**	0.86	**0.31**	**3.86 × 10^−16^**	0.78	0.44 (0.38, 0.51)
Grapefruit	stachydrine	Xenobiotics	**0.26**	**2.86 × 10^−11^**	0.73	0.19	1.63 × 10^−6^	0.62	0.50 (0.44, 0.55)
	3-hydroxystachydrine *	Xenobiotics	**0.22**	**1.34 × 10^−8^**	0.70	0.18	3.73 × 10^−6^	0.62	0.33 (0.27, 0.40)
Watermelon	X-25271		**0.37**	**2.65 × 10^−22^**	0.83	**0.26**	**1.47 × 10^−11^**	0.66	0.33 (0.27, 0.40)
Cantaloupe	X-25271		**0.30**	**6.08 × 10^−15^**	0.76	0.19	1.39 × 10^−6^	0.65	0.33 (0.27, 0.40)
Berries	methyl glucopyranoside (α + β )	Xenobiotics	0.17	2.00 × 10^−5^	0.83	**0.23**	**4.29 × 10^−9^**	0.76	0.62 (0.57, 0.67)
	X-24475		**0.21**	**7.82 × 10^−8^**	0.83	**0.21**	**7.16 × 10^−8^**	0.75	0.56 (0.51, 0.61)
	X-17354		0.16	2.32 × 10^−5^	0.83	**0.20**	**2.18 × 10^−7^**	0.73	0.62 (0.57, 0.67)
Blueberries	4-allylphenol sulfate	Xenobiotics	**0.22**	**1.24 × 10^−8^**	0.82	0.15	1.75 × 10^−4^	0.75	0.44 (0.38, 0.50)
	γ-tocopherol/ β -tocopherol	Cofactors and Vitamins	−0.15	1.72 × 10^−4^	0.80	**−0.22**	**1.62 × 10^−8^**	0.75	0.69 (0.65, 0.73)
	methyl glucopyranoside (α + β )	Xenobiotics	0.12	1.47 × 10^−3^	0.80	**0.23**	**2.21 × 10^−9^**	0.75	0.62 (0.57, 0.67)
Raspberries	methyl glucopyranoside (α + β )	Xenobiotics	0.16	4.89 × 10^−5^	0.77	**0.20**	**1.58 × 10^−7^**	0.65	0.62 (0.57, 0.67)
Peaches or plums	β -cryptoxanthin	Cofactors and Vitamins	0.18	3.25 × 10^−6^	0.78	**0.23**	**4.64 × 10^−9^**	0.73	0.77 (0.74, 0.80)
	X-12306		0.09	2.73 × 10^−2^	0.75	0.21	8.44 × 10^−8^	0.71	0.47 (0.41, 0.53)
**VEGETABLES**									
Tomatoes	4-hydroxychlorothalonil	Xenobiotics	**0.21**	**6.64 × 10^−8^**	0.82	0.15	8.00 × 10^−5^	0.72	0.85 (0.83, 0.87)
Asparagus	ergothioneine	Xenobiotics	**0.23**	**1.31 × 10^−9^**	0.77	0.12	1.67 × 10^−3^	0.62	0.86 (0.84, 0.88)
	X-11849		**0.20**	**1.63 × 10^−7^**	0.75	0.06	1.20 × 10^−1^	0.61	0.66 (0.62, 0.70)
	X-11847		**0.22**	**1.33 × 10^−8^**	0.75	0.07	9.24 × 10^−2^	0.60	0.58 (0.52, 0.63)
Beans	S-methylcysteine	Amino Acid	**0.21**	**3.85 × 10^−8^**	0.90	0.18	2.53 × 10^−6^	0.71	0.36 (0.30, 0.43)
	pipecolate	Amino Acid	**0.21**	**5.06 × 10^−8^**	0.89	0.19	1.68 × 10^−6^	0.72	0.32 (0.26, 0.39)
	X-11849		0.08	3.98 × 10^−2^	0.89	**0.21**	**5.75 × 10^−8^**	0.72	0.66 (0.62, 0.70)
Soy products	X-16649		**0.33**	**1.41 × 10^−18^**	0.77	**0.37**	**6.46 × 10^−23^**	0.75	0.46 (0.40, 0.52)
	X-24637		**0.33**	**3.68 × 10^−18^**	0.77	**0.36**	**8.49 × 10^−22^**	0.74	0.39 (0.33, 0.46)
	4-ethylphenyl sulfate	Xenobiotics	**0.30**	**4.86 × 10^−15^**	0.75	**0.35**	**1.37 × 10^−20^**	0.74	0.52 (0.47, 0.58)
Fermented soy products	X-11381		**−0.21**	**6.92 × 10^−8^**	0.66	−0.18	3.67 × 10^−6^	0.58	0.92 (0.91, 0.93)
	X-14939		−0.01	8.53 × 10^−1^	0.64	**−0.21**	**6.91 × 10^−8^**	0.59	0.68 (0.63, 0.72)
	X-11261		−0.07	7.02 × 10^−2^	0.64	**−0.22**	**6.49 × 10^−9^**	0.59	0.65 (0.60, 0.69)
Soymilk	4-ethylphenyl sulfate	Xenobiotics	**0.28**	**2.10 × 10^−13^**	0.65	**0.34**	**6.36 × 10^−19^**	0.64	0.52 (0.47, 0.58)
	X-24637		**0.26**	**5.13 × 10^−12^**	0.63	**0.33**	**1.23 × 10^−18^**	0.62	0.39 (0.33, 0.46)
	X-16649		**0.29**	**1.67 × 10^−14^**	0.62	**0.29**	**5.38 × 10^−14^**	0.62	0.46 (0.40, 0.52)
Soy protein powder	X-16649		**0.21**	**8.91 × 10^−8^**	0.63	0.13	1.06 × 10^−3^	0.60	0.46 (0.40, 0.52)
Cruciferous vegetables	S-methylcysteine	Amino Acid	**0.26**	**1.95 × 10^−11^**	0.85	0.14	3.61 × 10^−4^	0.74	0.36 (0.30, 0.43)
	carotene diol (2)	Cofactors and Vitamins	**0.23**	**1.78 × 10^−9^**	0.83	**0.20**	**1.35 × 10^−7^**	0.74	0.79 (0.75, 0.81)
	X-13866		**0.26**	**2.71 × 10^−11^**	0.83	0.12	2.27 × 10^−3^	0.71	0.52 (0.47, 0.58)
Leafy greens	carotene diol (1)	Cofactors and Vitamins	**0.23**	**1.56 × 10^−9^**	0.84	**0.23**	**1.28 × 10^−9^**	0.76	0.83 (0.80, 0.85)
	carotene diol (2)	Cofactors and Vitamins	**0.22**	**2.02 × 10^−8^**	0.83	**0.21**	**3.22 × 10^−8^**	0.75	0.79 (0.75, 0.81)
	docosahexaenoate (DHA; 22:6 n3)	Lipid	**0.20**	**2.24 × 10^−7^**	0.81	0.12	2.02 × 10^−3^	0.69	0.55 (0.50, 0.60)
Iceberg or head lettuce	pentose acid *	Partially Characterized Molecules	**−0.23**	**1.09 × 10^−9^**	0.71	−0.03	4.61 × 10^−1^	0.57	0.56 (0.50, 0.61)
Peppers	X-23780		**0.29**	**3.19 × 10^−14^**	0.81	0.18	4.96 × 10^−6^	0.75	0.39 (0.33, 0.46)
Mushrooms ^4^	ergothioneine	Xenobiotics				**0.26**	**2.57 × 10^−11^**	0.70	0.86 (0.84, 0.88)
	X-11847					**0.24**	**6.54 × 10^−10^**	0.69	0.58 (0.52, 0.63)
	X-11858					**0.22**	**1.34 × 10^−8^**	0.69	0.52 (0.47, 0.58)
Allium vegetables	N-methyltaurine	Amino Acid	**0.27**	**3.08 × 10^−12^**	0.81	0.20	4.41 × 10^−7^	0.73	0.32 (0.25, 0.39)
	ergothioneine	Xenobiotics	**0.22**	**1.18 × 10^−8^**	0.80	0.10	7.38 × 10^−3^	0.71	0.86 (0.84, 0.88)
	N-acetylalliin	Xenobiotics	**0.22**	**1.19 × 10^−8^**	0.79	0.06	1.08 × 10^−1^	0.70	0.29 (0.22, 0.36)
Onion	N-methyltaurine	Amino Acid	**0.26**	**5.25 × 10^−12^**	0.82	0.19	1.04 × 10^−6^	0.72	0.32 (0.25, 0.39)
	ergothioneine	Xenobiotics	**0.21**	**4.36 × 10^−8^**	0.79	0.10	1.08 × 10^−2^	0.70	0.86 (0.84, 0.88)
	N-acetylalliin	Xenobiotics	**0.21**	**1.07 × 10^−7^**	0.79	0.06	1.57 × 10^−1^	0.69	0.29 (0.22, 0.36)
Garlic	N-methyltaurine	Amino Acid	**0.25**	**8.60 × 10^−11^**	0.81	**0.24**	**8.70 × 10^−10^**	0.74	0.32 (0.25, 0.39)
	δ-CEHC	Cofactors and Vitamins	**−0.23**	**3.48 × 10^−9^**	0.81	−0.14	4.34 × 10^−4^	0.69	0.48 (0.42, 0.54)
	N-acetylalliin	Xenobiotics	**0.29**	**3.06 × 10^−14^**	0.81	0.12	2.72 × 10^−3^	0.67	0.29 (0.22, 0.36)
Garlic powder	S-allylcysteine	Xenobiotics	**0.22**	**1.25 × 10^−8^**	0.74	0.08	5.13 × 10^−2^	0.68	0.31 (0.24, 0.38)
**GRAINS**									
Whole grains	X-21752		**0.31**	**8.54 × 10^−16^**	0.89	0.19	1.10 × 10^−6^	0.80	0.71 (0.67, 0.75)
	2,6-dihydroxybenzoic acid	Xenobiotics	**0.23**	**1.22 × 10^−9^**	0.88	0.18	3.38 × 10^−6^	0.79	0.62 (0.57, 0.67)
	4-methoxyphenol sulfate	Amino Acid	**0.21**	**9.90 × 10^−8^**	0.87	0.17	8.86 × 10^−6^	0.77	0.34 (0.28, 0.41)
Whole grain bread	2-aminophenol sulfate	Xenobiotics	**0.22**	**7.79 × 10^−9^**	0.80	0.20	4.50 × 10^−7^	0.71	0.45 (0.39, 0.51)
Whole grain cereals	X-21752		**0.42**	**7.24 × 10^−29^**	0.87	**0.38**	**2.86 × 10^−24^**	0.84	0.71 (0.67, 0.75)
	2,6-dihydroxybenzoic acid	Xenobiotics	**0.27**	**1.50 × 10^−12^**	0.80	**0.22**	**1.69 × 10^−8^**	0.79	0.62 (0.57, 0.67)
	2-aminophenol sulfate	Xenobiotics	**0.30**	**6.86 × 10^−15^**	0.79	**0.25**	**5.65 × 10^−11^**	0.80	0.45 (0.39, 0.51)
Corn products	X-24545		**0.23**	**2.55 × 10^−9^**	0.83	0.08	4.03 × 10^−2^	0.71	0.72 (0.68, 0.75)
	X-16935		**0.21**	**5.49 × 10^−8^**	0.83	0.15	7.12 × 10^−5^	0.71	0.89 (0.87, 0.90)
	γ-tocopherol/ β -tocopherol	Cofactors and Vitamins	**0.20**	**1.84 × 10^−7^**	0.83	0.02	5.57 × 10^−1^	0.71	0.69 (0.65, 0.73)
Refined grains	γ-tocopherol/ β -tocopherol	Cofactors and Vitamins	**0.24**	**4.12 × 10^−10^**	0.84	0.12	1.86 × 10^−3^	0.85	0.69 (0.65, 0.73)
	X-24475		**−0.20**	**1.29 × 10^−7^**	0.84	−0.17	1.19 × 10^−5^	0.84	0.56 (0.51, 0.61)
	X-23680		0.13	9.26 × 10^−4^	0.83	**0.21**	**4.76 × 10^−8^**	0.85	0.57 (0.52, 0.62)
**PROTEINS**									
Eggs	PE (*p*-18:0/20:4) *	Lipid	**0.25**	**5.58 × 10^−11^**	0.79	**0.20**	**3.60 × 10^−7^**	0.75	0.68 (0.63, 0.72)
	PE (*p*-16:0/20:4) *	Lipid	**0.21**	**8.46 × 10^−8^**	0.78	0.18	2.67 × 10^−6^	0.73	0.60 (0.55, 0.65)
Red meat	X-11381		**0.40**	**2.62 × 10^−26^**	0.88	**0.37**	**1.28 × 10^−22^**	0.83	0.92 (0.91, 0.93)
	PE (*p*-18:0/20:4) *	Lipid	**0.40**	**4.29 × 10^−26^**	0.88	**0.37**	**1.73 × 10^−22^**	0.82	0.68 (0.63, 0.72)
	PE (*p*-18:0/18:1)	Lipid	**0.30**	**1.61 × 10^−15^**	0.87	**0.26**	**3.22 × 10^−11^**	0.79	0.54 (0.49, 0.59)
Processed meat	PE (*p*-18:0/20:4) *	Lipid	**0.38**	**2.27 × 10^−23^**	0.85	**0.31**	**6.65 × 10^−16^**	0.80	0.68 (0.63, 0.72)
	PE (*p*-16:0/20:4) *	Lipid	**0.31**	**1.03 × 10^−15^**	0.83	**0.30**	**5.97 × 10^−15^**	0.80	0.60 (0.55, 0.65)
	PC (*p*-16:0/20:4) *	Lipid	**0.31**	**8.70 × 10^−16^**	0.83	**0.24**	**8.37 × 10^−10^**	0.78	0.73 (0.70, 0.77)
Poultry	PE (*p*-16:0/20:4) *	Lipid	**0.47**	**3.24 × 10^−37^**	0.87	**0.42**	**6.64 × 10^−30^**	0.83	0.60 (0.55, 0.65)
	PE (*p*-18:0/20:4) *	Lipid	**0.45**	**2.97 × 10^−34^**	0.85	**0.40**	**4.40 × 10^−27^**	0.81	0.68 (0.63, 0.72)
	3-methylhistidine	Amino Acid	**0.54**	**5.73 × 10^−51^**	0.85	**0.40**	**8.50 × 10^−27^**	0.81	0.45 (0.39, 0.51)
Total fish	hydroxy-CMPF *	Lipid	**0.43**	**1.37 × 10^−31^**	0.84	**0.27**	**8.43 × 10^−13^**	0.72	0.96 (0.95, 0.96)
	CMPF	Lipid	**0.43**	**1.94 × 10^−30^**	0.83	**0.30**	**1.31 × 10^−15^**	0.73	0.86 (0.84, 0.88)
	PC (16:0/22:6)	Lipid	**0.30**	**1.52 × 10^−15^**	0.81	**0.27**	**3.03 × 10^−12^**	0.71	0.77 (0.74, 0.80)
Dark meat fish	hydroxy-CMPF *	Lipid	**0.44**	**3.03 × 10^−32^**	0.85	**0.27**	**2.07 × 10^−12^**	0.74	0.96 (0.95, 0.96)
	CMPF	Lipid	**0.43**	**2.93 × 10^−31^**	0.84	**0.28**	**1.58 × 10^−13^**	0.75	0.86 (0.84, 0.88)
	PC (16:0/22:6)	Lipid	**0.35**	**4.59 × 10^−20^**	0.83	**0.24**	**7.08 × 10^−10^**	0.72	0.77 (0.74, 0.80)
Shellfish	X-25810		**0.35**	**2.61 × 10^−20^**	0.77	**0.24**	**3.13 × 10^−10^**	0.70	0.55 (0.50, 0.60)
	CMPF	Lipid	**0.27**	**1.28 × 10^−12^**	0.74	0.17	7.56 × 10^−6^	0.70	0.86 (0.84, 0.88)
	X-25419		**0.36**	**9.16 × 10^−22^**	0.73	**0.20**	**2.06 × 10^−7^**	0.69	0.64 (0.60, 0.69)
Total nuts	tryptophan betaine	Amino Acid	**0.43**	**8.29 × 10^−31^**	0.91	**0.30**	**4.82 × 10^−15^**	0.83	0.82 (0.80, 0.85)
	X-11315		**0.27**	**1.22 × 10^−12^**	0.91	**0.26**	**1.62 × 10^−11^**	0.82	0.64 (0.59, 0.68)
	X-23644		**0.31**	**5.97 × 10^−16^**	0.89	**0.26**	**1.11 × 10^−11^**	0.80	0.32 (0.26, 0.39)
Peanuts	4-vinylphenol sulfate	Xenobiotics	**0.39**	**1.27 × 10^−25^**	0.87	**0.23**	**4.54 × 10^−9^**	0.70	0.39 (0.32, 0.46)
	tryptophan betaine	Amino Acid	**0.39**	**7.63 × 10^−26^**	0.86	**0.33**	**1.14 × 10^−17^**	0.77	0.82 (0.80, 0.85)
	behenoylcarnitine (C22) *	Lipid	**0.33**	**2.54 × 10^−18^**	0.85	**0.20**	**1.76 × 10^−7^**	0.69	0.45 (0.39, 0.51)
Other nuts	X-11315		**0.29**	**1.26 × 10^−14^**	0.89	**0.32**	**3.85 × 10^−17^**	0.84	0.64 (0.59, 0.68)
	X-24475		**0.30**	**2.21 × 10^−15^**	0.87	**0.30**	**7.54 × 10^−15^**	0.82	0.56 (0.51, 0.61)
	tryptophan betaine	Amino Acid	**0.25**	**9.08 × 10^−11^**	0.85	0.19	7.64 × 10^−7^	0.78	0.82 (0.80, 0.85)
Seeds	X-11858		0.17	1.89 × 10^−5^	0.75	**0.27**	**4.08 × 10^−12^**	0.76	0.52 (0.47, 0.58)
	ergothioneine	Xenobiotics	**0.23**	**3.15 × 10^−9^**	0.75	**0.21**	**5.22 × 10^−8^**	0.72	0.86 (0.84, 0.88)
	X-17354		**0.21**	**6.02 × 10^−8^**	0.74	**0.26**	**1.57 × 10^−11^**	0.75	0.62 (0.57, 0.67)
**DAIRY/DAIRY ALTERNATIVES**									
Milk	X-11381		**0.33**	**3.73 × 10^−18^**	0.84	**0.27**	**3.03 × 10^−12^**	0.77	0.92 (0.91, 0.93)
	N,N,N-trimethyl-5-aminovalerate	Amino Acid	**0.27**	**2.10 × 10^−12^**	0.83	**0.23**	**4.07 × 10^−9^**	0.73	0.87 (0.85, 0.89)
	3-bromo-5-chloro-2,6-dihydroxybenzoic acid *	Xenobiotics	**0.28**	**3.04 × 10^−13^**	0.82	**0.23**	**1.36 × 10^−9^**	0.75	0.75 (0.72, 0.79)
Almond or rice milk	X-24475		**0.24**	**4.75 × 10^−10^**	0.72	0.19	1.59 × 10^−6^	0.65	0.56 (0.51, 0.61)
	3-bromo-5-chloro-2,6-dihydroxybenzoic acid *	Xenobiotics	−0.18	3.89 × 10^−6^	0.71	**−** **0.21**	**3.93 × 10^−8^**	0.64	0.75 (0.72, 0.79)
	3,5-dichloro-2,6-dihydroxybenzoic acid	Xenobiotics	−0.19	1.39 × 10^−6^	0.70	**−0.21**	**1.05 × 10^−7^**	0.64	0.89 (0.88, 0.91)
Total cheese	heptenedioate (C7:1-DC) *	Lipid	**0.30**	**1.58 × 10^−15^**	0.88	**0.23**	**1.28 × 10^−9^**	0.78	0.44 (0.38, 0.50)
	SM (d17:2/16:0, d18:2/15:0) *	Lipid	**0.24**	**2.57 × 10^−10^**	0.88	0.19	1.80 × 10^−6^	0.78	0.65 (0.60, 0.69)
	margaroylcarnitine (C17) *	Lipid	**0.25**	**1.24 × 10^−10^**	0.88	**0.20**	**1.21 × 10^−7^**	0.78	0.37 (0.31, 0.44)
Cream	X-21442		**0.36**	**2.77 × 10^−21^**	0.80	0.12	3.11 × 10^−3^	0.71	0.87 (0.85, 0.88)
	quinate	Xenobiotics	**0.34**	**1.82 × 10^−19^**	0.80	0.12	2.97 × 10^−3^	0.70	0.81 (0.79, 0.84)
	X-12816		**0.26**	**2.67 × 10^−11^**	0.75	0.11	4.69 × 10^−3^	0.70	0.87 (0.85, 0.89)
**FATS AND OILS**									
Creamy salad dressing	X-16944		**0.27**	**1.69 × 10^−12^**	0.78	**0.25**	**7.40 × 10^−11^**	0.70	0.59 (0.54, 0.64)
	X-11261		**0.28**	**2.55 × 10^−13^**	0.78	**0.22**	**1.92 × 10^−8^**	0.69	0.65 (0.60, 0.69)
	X-15486		**0.27**	**1.40 × 10^−12^**	0.78	**0.20**	**1.22 × 10^−7^**	0.68	0.55 (0.49, 0.60)
Oil and vinegar salad dressing	carotene diol (1)	Cofactors and Vitamins	0.18	4.41 × 10^−6^	0.76	**0.22**	**1.25 × 10^−8^**	0.81	0.83 (0.80, 0.85)
	X-24475		**0.22**	**1.50 × 10^−8^**	0.76	0.09	2.84 × 10^−2^	0.78	0.56 (0.51, 0.61)
Olive oil	X-25419		**0.22**	**1.57 × 10^−8^**	0.78	0.15	8.26 × 10^−5^	0.74	0.64 (0.60, 0.69)
	δ-CEHC	Cofactors and Vitamins	**−0.24**	**2.84 × 10^−10^**	0.78	−0.15	1.87 × 10^−4^	0.74	0.48 (0.42, 0.54)
**MISCELLANEOUS**									
French fries	γ-tocopherol/ β -tocopherol	Cofactors and Vitamins	**0.21**	**4.60 × 10^−8^**	0.85	0.10	1.48 × 10^−2^	0.72	0.69 (0.65, 0.73)
	pentose acid *	Partially Characterized Molecules	**−0.24**	**4.27 × 10^−10^**	0.85	−0.07	6.36 × 10^−2^	0.71	0.56 (0.50, 0.61)
	X-07765		**0.23**	**4.00 × 10^−9^**	0.84	0.07	7.28 × 10^−2^	0.71	0.47 (0.41, 0.53)
Ice cream	X-07765		**0.20**	**1.43 × 10^−7^**	0.82	0.09	1.95 × 10^−2^	0.68	0.47 (0.41, 0.53)
	tridecenedioate (C13:1-DC) *	Lipid	**0.21**	**7.05 × 10^−8^**	0.80	0.07	6.05 × 10^−2^	0.68	0.58 (0.53, 0.63)
	margaroylcarnitine (C17) *	Lipid	**0.21**	**8.68 × 10^−8^**	0.80	0.10	1.19 × 10^−2^	0.68	0.37 (0.31, 0.44)
Chips	X-21339		**0.31**	**1.57 × 10^−16^**	0.81	**0.26**	**1.38 × 10^−11^**	0.78	0.90 (0.89, 0.92)
	X-11880		**0.30**	**1.04 × 10^−14^**	0.80	**0.28**	**1.35 × 10^−13^**	0.80	0.90 (0.89, 0.91)
	X-11308		**0.25**	**1.29 × 10^−10^**	0.79	0.19	1.65 × 10^−6^	0.76	0.95 (0.95, 0.96)
Chocolate candies	X-13728		**0.32**	**1.62 × 10^−17^**	0.83	**0.32**	**1.11 × 10^−16^**	0.84	0.54 (0.48, 0.59)
	theobromine	Xenobiotics	**0.29**	**1.39 × 10^−14^**	0.82	**0.29**	**5.24 × 10^−14^**	0.83	0.56 (0.51, 0.62)
	3,7-dimethylurate	Xenobiotics	**0.29**	**1.46 × 10^−14^**	0.82	**0.29**	**1.82 × 10^−14^**	0.83	0.46 (0.40, 0.52)
Dark chocolate	theobromine	Xenobiotics	**0.26**	**7.78 × 10^−12^**	0.80	**0.22**	**1.64 × 10^−8^**	0.72	0.56 (0.51, 0.62)
	X-13728		**0.30**	**1.70 × 10^−15^**	0.80	**0.22**	**5.69 × 10^−9^**	0.71	0.54 (0.48, 0.59)
	7-methylxanthine	Xenobiotics	**0.29**	**3.17 × 10^−14^**	0.79	**0.23**	**3.73 × 10^−9^**	0.72	0.48 (0.42, 0.54)
Energy/protein Bars	X-16649		**0.20**	**1.40 × 10^−7^**	0.80	0.19	1.79 × 10^−6^	0.71	0.46 (0.40, 0.52)
Soy sauce	X-11858		**0.20**	**2.42 × 10^−7^**	0.74	**0.21**	**1.09 × 10^−7^**	0.66	0.52 (0.47, 0.58)
	X-11849		**0.20**	**2.33 × 10^−7^**	0.74	**0.20**	**1.53 × 10^−7^**	0.66	0.66 (0.62, 0.70)
	X-11847		**0.20**	**1.59 × 10^−7^**	0.74	**0.20**	**1.47 × 10^−7^**	0.66	0.58 (0.52, 0.63)
Artificial sweeteners	acesulfame	Xenobiotics	**0.24**	**3.28 × 10^−10^**	0.75	**0.23**	**2.16 × 10^−9^**	0.74	0.49 (0.43, 0.55)
	saccharin	Xenobiotics	**0.21**	**9.48 × 10^−8^**	0.68	**0.21**	**3.09 × 10^−8^**	0.66	0.59 (0.54, 0.64)
	erythritol	Xenobiotics	0.19	1.07 × 10^−6^	0.66	**0.20**	**1.28 × 10^−7^**	0.63	0.48 (0.42, 0.54)
**ALCOHOL**									
Total alcohol	ethyl α-glucopyranoside	Xenobiotics	**0.52**	**9.89 × 10^−47^**	0.94	**0.46**	**2.91 × 10^−36^**	0.88	0.52 (0.47, 0.58)
	ethyl glucuronide	Xenobiotics	**0.43**	**1.11 × 10^−31^**	0.92	**0.40**	**3.66 × 10^−27^**	0.87	0.57 (0.52, 0.62)
	2,3-dihydroxyisovalerate	Xenobiotics	**0.37**	**2.51 × 10^−23^**	0.91	**0.40**	**1.57 × 10^−26^**	0.85	0.46 (0.40, 0.52)
Beer	ethyl α-glucopyranoside	Xenobiotics	**0.38**	**1.23 × 10^−23^**	0.84	**0.33**	**7.63 × 10^−18^**	0.82	0.52 (0.47, 0.58)
	theophylline	Xenobiotics	**0.29**	**1.18 × 10^−14^**	0.82	**0.23**	**2.04 × 10^−9^**	0.81	0.78 (0.74, 0.81)
	X-11795		**0.25**	**3.27 × 10^−11^**	0.82	**0.23**	**1.13 × 10^−9^**	0.81	0.56 (0.51, 0.62)
Total wine	ethyl α-glucopyranoside	Xenobiotics	**0.49**	**6.42 × 10^−41^**	0.91	**0.41**	**8.57 × 10^−28^**	0.80	0.52 (0.47, 0.58)
	2,3-dihydroxyisovalerate	Xenobiotics	**0.44**	**1.69 × 10^−32^**	0.86	**0.42**	**7.57 × 10^−30^**	0.80	0.46 (0.40, 0.52)
	ethyl glucuronide	Xenobiotics	**0.45**	**6.20 × 10^−34^**	0.85	**0.40**	**1.30 × 10^−26^**	0.82	0.57 (0.52, 0.62)
Red wine	ethyl α-glucopyranoside	Xenobiotics	**0.45**	**1.76 × 10^−33^**	0.83	**0.35**	**1.05 × 10^−20^**	0.78	0.52 (0.47, 0.58)
	2,3-dihydroxyisovalerate	Xenobiotics	**0.40**	**2.37 × 10^−27^**	0.79	**0.36**	**2.34 × 10^−21^**	0.77	0.46 (0.40, 0.52)
	pentose acid *	Partially Characterized Molecules	**0.32**	**3.25 × 10^−17^**	0.79	**0.31**	**1.17 × 10^−15^**	0.76	0.56 (0.50, 0.61)
White wine	ethyl α-glucopyranoside	Xenobiotics	**0.33**	**1.17 × 10^−18^**	0.73	**0.32**	**2.76 × 10^−17^**	0.72	0.52 (0.47, 0.58)
	pentose acid *	Partially Characterized Molecules	**0.24**	**5.40 × 10^−10^**	0.71	**0.29**	**2.21 × 10^−14^**	0.74	0.56 (0.50, 0.61)
	X-11795		**0.25**	**1.17 × 10^−10^**	0.70	**0.25**	**1.27 × 10^−10^**	0.69	0.56 (0.51, 0.62)
Liquor	ethyl α-glucopyranoside	Xenobiotics	**0.34**	**6.55 × 10^−19^**	0.75	0.16	2.35 × 10^−5^	0.68	0.52 (0.47, 0.58)
	2-hydroxyphytanate *	Lipid	**0.20**	**1.25 × 10^−7^**	0.72	0.14	4.17 × 10^−4^	0.67	0.60 (0.55, 0.65)
	ethyl glucuronide	Xenobiotics	**0.30**	**3.51 × 10^−15^**	0.72	0.15	1.80 × 10^−4^	0.66	0.57 (0.52, 0.62)
**BEVERAGES**									
Total coffee	X-21442		**0.81**	**0.35 × 10^−153^**	0.99	**0.80**	**0.81 × 10^−148^**	0.99	0.87 (0.85, 0.88)
	quinate	Xenobiotics	**0.77**	**0.38 × 10^−129^**	0.99	**0.74**	**0.13 × 10^−113^**	0.97	0.81 (0.79, 0.84)
	X-23655		**0.56**	**4.11 × 10^−56^**	0.98	**0.52**	**3.17 × 10^−47^**	0.95	0.64 (0.59, 0.68)
Decaffeinated	X-21442		**0.27**	**1.09 × 10^−12^**	0.70	**0.23**	**1.20 × 10^−9^**	0.65	0.87 (0.85, 0.88)
	quinate	Xenobiotics	**0.21**	**1.08 × 10^−7^**	0.69	0.15	1.43 × 10^−4^	0.65	0.81 (0.79,0.84)
Caffeinated	X-21442		**0.75**	**0.21 × 10^−120^**	0.98	**0.75**	**0.57 × 10^−118^**	0.97	0.87 (0.85, 0.88)
	quinate	Xenobiotics	**0.71**	**0.96 × 10^−101^**	0.98	**0.71**	**0.81 × 10^−100^**	0.97	0.81 (0.79, 0.84)
	3-hydroxypyridine sulfate	Xenobiotics	**0.59**	**3.37 × 10^−63^**	0.96	**0.57**	**5.09 × 10^−57^**	0.94	0.72 (0.69, 0.76)
Total tea	theanine	Xenobiotics	**0.40**	**8.58 × 10^−27^**	0.86	**0.39**	**1.53 × 10^−25^**	0.83	0.60 (0.55, 0.65)
	X-17685		**0.20**	**2.32 × 10^−7^**	0.73	**0.24**	**5.72 × 10^−10^**	0.74	0.50 (0.44, 0.55)
	3-methoxycatechol sulfate (1)	Xenobiotics	**0.20**	**2.49 × 10^−7^**	0.73	**0.22**	**1.46 × 10^−8^**	0.72	0.42 (0.36, 0.49)
Green tea	theanine	Xenobiotics	**0.25**	**5.18 × 10^−11^**	0.72	**0.28**	**1.44 × 10^−13^**	0.69	0.60 (0.55, 0.65)
Black tea	theanine	Xenobiotics	**0.34**	**1.03 × 10^−19^**	0.76	**0.35**	**8.60 × 10^−21^**	0.77	0.60 (0.55, 0.65)
	X-17685		**0.23**	**2.89 × 10^−9^**	0.69	**0.20**	**1.66 × 10^−7^**	0.68	0.50 (0.44, 0.55)
	3-methoxycatechol sulfate (1)	Xenobiotics	**0.20**	**1.68 × 10^−7^**	0.68	0.19	1.74 × 10^−6^	0.66	0.42 (0.36, 0.49)
Herbal tea	X-18901		**0.22**	**9.41 × 10^−9^**	0.74	**0.20**	**2.60 × 10^−7^**	0.65	0.68 (0.63, 0.72)
	X-12306		**0.20**	**1.50 × 10^−7^**	0.71	0.18	4.49 × 10^−6^	0.63	0.47 (0.41, 0.53)
Diet beverages	acesulfame	Xenobiotics	**0.42**	**1.15 × 10^−29^**	0.82	**0.29**	**4.97 × 10^−14^**	0.76	0.49 (0.43, 0.55)

^1^. Diet–metabolite correlations in **bold** had *p* < 4.33 × 10^−7^ for FFQ and *p* < 4.17 × 10^−7^ for 24-h diet recalls and |*r*| > 0.2 from Pearson’s partial correlation analysis. Adjusted for age, gender, race/ethnicity, education, smoking status, physical activity, body mass index, ethanol consumption (except for alcohol-containing items), and energy intake. CPS-3, Cancer Prevention Study-3; DAS, Diet Assessment Sub-study. ^2^. Biochemical name of metabolite correlated with respective food or food group. Metabolites starting with X are unnamed and the super pathway of these is unknown. Asterisk (*) represents putative identity that has not been officially confirmed based on a standard. (1) and (2) indicate that the metabolite differs from another with the same mass in the position of the R group. CMPF, 3-carboxy-4-methyl-5-propyl-2-furanpropanoate; PC, phosphatidylcholine; PE, phosphatidylethanolamine. ^3^. ICC, intraclass correlation coefficient, to assess the reproducibility of the identified food-related metabolites over six months. ^4^. Items are only available on 24 h-diet recalls.

## Supplementary Materials

The following are available online at https://www.mdpi.com/2218-1989/10/10/382/s1. Figure S1: study population exclusion; Table S1: food–metabolite associations identified using either FFQ or 24-h diet recalls in the CPS-3 Diet Assessment Sub-study; Table S2: food–metabolite associations identified using the FFQ in the CPS-3 Diet Assessment Sub-study; Table S3: Food–metabolite associations identified using the average 24-h diet recalls in the CPS-3 Diet Assessment Sub-study; Table S4: food group definitions in the CPS-3 Diet Assessment Sub-study.
