# Supplementary material for: Identification and Reproducibility of Plasma Metabolomic Biomarkers of Habitual Food Intake in a US Diet Validation Study
Source: Metabolites. 2020 Sep 26;10(10):382. doi: 10.3390/metabo10100382 (PMC7600452; doi:10.3390/metabo10100382)
Supplement: Supplementary file 1 [file metabolites-10-00382-s001.zip › Figure S1.pdf]

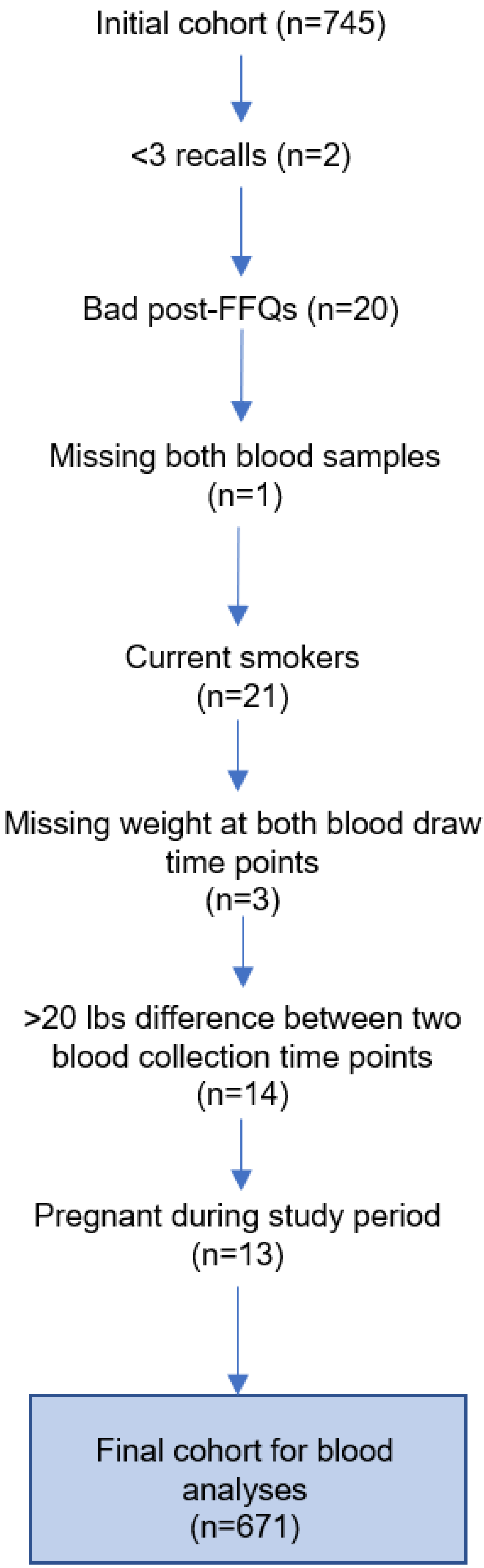
Initial cohort (n=745)
<3 recalls (n=2)
Bad post-FFQs (n=20)
Missing both blood samples (n=1)
Current smokers (n=21)
Missing weight at both blood draw time points (n=3)
>20 lbs difference between two blood collection time points (n=14)
Pregnant during study period (n=13)
Final cohort for blood analyses (n=671)
